# Supplementary material for: Streptococcus pneumoniae Evades Host Cell Phagocytosis and Limits Host Mortality Through Its Cell Wall Anchoring Protein PfbA
Source: Front Cell Infect Microbiol. 2019 Aug 20;9:301. doi: 10.3389/fcimb.2019.00301 (PMC6710382; doi:10.3389/fcimb.2019.00301)
Supplement: Supplementary file 1 [file Data_Sheet_1.pdf]

Supplementary Information

***Streptococcus pneumoniae* evades host cell phagocytosis and limits host mortality through its cell wall anchoring protein PfbA**

Running title: PfbA inhibits phagocytosis and limits host responses

Masaya Yamaguchi[a,#], Yujiro Hirose[a], Moe Takemura[a, b], Masayuki Ono[a, c], Tomoko Sumitomo[a], Masanobu Nakata[a], Yutaka Terao[d], Shigetada Kawabata[a #]

[a]Department of Oral and Molecular Microbiology, Osaka University Graduate School of Dentistry, Osaka, Japan

[b]Department of Oral and Maxillofacial Surgery II, Osaka University Graduate School of Dentistry, Osaka, Japan

[c]Department of Fixed Prosthodontics, Osaka University Graduate School of Dentistry, Osaka, Japan.

[d]Division of Microbiology and Infectious Diseases, Niigata University Graduate School of Medical and Dental Sciences, Niigata, Japan

[#]Address correspondence to Masaya Yamaguchi, yamaguchi@dent.osaka-u.ac.jp and Shigetada Kawabata, kawabata@dent.osaka-u.ac.jp

Supplementary text

Stimulation of the human monocytic cell line THP1 by a TLR ligand, LPS, induces miR-146a/b expression in an NF-κB-dependent fashion, and this induction inhibits innate immune responses (Taganov et al., 2006). Additionally, pneumococcal infection of human macrophages induces expression of several microRNAs (miRNAs), including miR-146a, in a TLR2-dependent manner, which prevents excessive inflammation (Griss et al., 2016). We performed miRNA array analysis using neutrophil-like differentiated HL60 cells, *S. pneumoniae* strains, and rPfbA (Supplementary Fig. 4; accession number: GSE128341) and compared rPfbA-treated and untreated cells, WT- and Δ*pfbA*-infected cells, and Δ*pfbA* with and without rPfbA-infected cells. The analysis revealed only one miRNA (hsa-miR-1281) commonly downregulated by ≥2-fold in the presence of PfbA as compared to results in its absence (Supplementary Fig. 4; magenta circle). On the other hand, there were no commonly upregulated miRNAs, including miR-146a/b. Moreover, the expression of eight miRNAs was commonly altered following WT or Δ*pfbA* infection and in the presence of rPfbA as compared with infection with Δ*pfbA* only. Five miRNAs (hsa-miR-4674, hsa-miR-3613-3p, hsa-miR-4668-5p, hsa-

miR-3197, and hsa-miR-6802-5p) were upregulated, whereas three (hsa-miR-3935, hsa-miR-1281, and hsa-miR-3613-5p) were downregulated. However, the role of these miRNAs in the infection process remains unclear.

Supplementary Reference

Griss, K., Bertrams, W., Sittka-Stark, A., Seidel, K., Stielow, C., Hippenstiel, S., et al. (2016). MicroRNAs Constitute a Negative Feedback Loop in *Streptococcus pneumoniae*-Induced Macrophage Activation. *J Infect Dis* 214(2), 288-299. doi: 10.1093/infdis/jiw109.

Taganov, K.D., Boldin, M.P., Chang, K.J., and Baltimore, D. (2006). NF-κB-dependent induction of microRNA miR-146, an inhibitor targeted to signaling proteins of innate immune responses. *Proc Natl Acad Sci U S A* 103(33), 12481-12486. doi: 10.1073/pnas.0605298103.

Supplementary Figure 1. Maximum likelihood phylogenetic analyses of the *pfbA* gene

The codon-based maximum likelihood phylogenetic relationship was calculated using the RAxML program. Strains with identical sequences are listed on the same branch. *S. pneumoniae* and *S. pseudopneumoniae pfbA* genes are shaded in cyan. Other mitis-group bacterial *pfbA* genes are shaded in magenta. *S. mitis pfbA* genes in *S. pneumoniae* are shaded in yellow. The bootstrap values are shown near the nodes. The scale bar indicates nucleotide substitutions per site.

Supplementary Figure 2. SEAPorter assay using TLR2/NF-κB/SEAPorter cell lines.

Cells were plated in 24-well plates at 5×10^5 cells/well, and after 24 h, stimulated with **A.** live or pasteurized *S. pneumoniae* (~5×10^5 CFU) or **B.** untreated or pasteurized rPfbA for 24 h. SEAP was analyzed using the SEAPorter assay kit. Data are presented as the mean of six wells. HK denotes pasteurized samples. Standard error values are represented by vertical lines. Differences in untreated and pasteurized groups and those using the same concentrations were analyzed using a Kruskal–Wallis test, followed by Dunn's multiple comparisons test, respectively.

Supplementary Figure 3. Mice were intravenously infected with *S. pneumoniae* TIGR4 wild-type or Δ*pfbA* strains.

A. Plasma samples were collected from intravenously infected mice at 48 hours after infection. Values are presented as the mean of 16 or 18 samples. Vertical lines represent the median ± IQR. Statistical differences between groups were analyzed using Mann-Whitney’s U test. **B.** The bacterial burden in the blood, brain, lung, and liver were assessed after 48 h of infection. The medians and IQR values are represented by vertical lines. All mice were perfused with PBS after blood collection, organ samples were collected. Statistical differences between groups were analyzed using Mann-Whitney's U test.

Supplementary Figure 4. microRNA array analysis of differentiated HL60 cells

miRNA array analysis was performed using the Affymetrix GeneChip® miRNA 4.0 array. Bacterial cells and/or rPfbA were incubated with differentiated HL-60 cells for 1 h at 37°C in a 5% CO_2 atmosphere. Total RNA including micro RNA was purified with an

miRNeasy kit. X and Y axes represent expression values after normalization. The red dashed line represents 2-fold change. Magenta circle means commonly downregulated miRNA by 2-fold or greater in the presence of PfbA as compared to in its absence.

Supplementary Table 1. Primers used in this study

Primers	Sequence (5' to 3')
For knockout plasmid construction	
T4pfbAKOuF	ccgcgggaattcgatgtgtcttgttctagttttcaattca
T4pfbAKOuR	tattcaaatatatcccatcagaacctccaattttttact
T4pfbAKOaF	ttggaggttctgatgggatatatttgaatacatacgaaca
T4pfbAKOaR	atttatctttaccattcaatttttttataattttttaat
T4pfbAKOdF	ttataaaaaaattgaatggtaaagataaataaaatttgtt
T4pfbAKOdR	gaattcactagtgattcaaacatcaatgactagaatactt
T4pfbAKOvF	gtcattgatgtttgaatcactagtgaattcgcggccgcct
T4pfbAKOvR	actagaacaagacacatcgaattcccgcggccgccatggc
For double-crossover recombination	
pfbAKOuMaxF	gtgtcttgttctagttttcaattca
pfbAKOdMaxR	tcaaacatcaatgactagaatactt
For mutation confirmation	
T4pfbAKOupup	tagcatttagaatccttactagac
T4pfbAKOdndn	ccccaatacgttcaatgtcagttg

Supplementary Table 2. The distribution of bacterial burden in mouse blood at 48 h after infection.

Bacterial burden	WT-infected group	Δ*pfbA*-infected group
>10^6 CFU	3	7
Between 10^6 and 10 CFU	11	6
Below detection limit	2	5
Total	16	18

Actual data is shown in Supplementary Figure 4B.

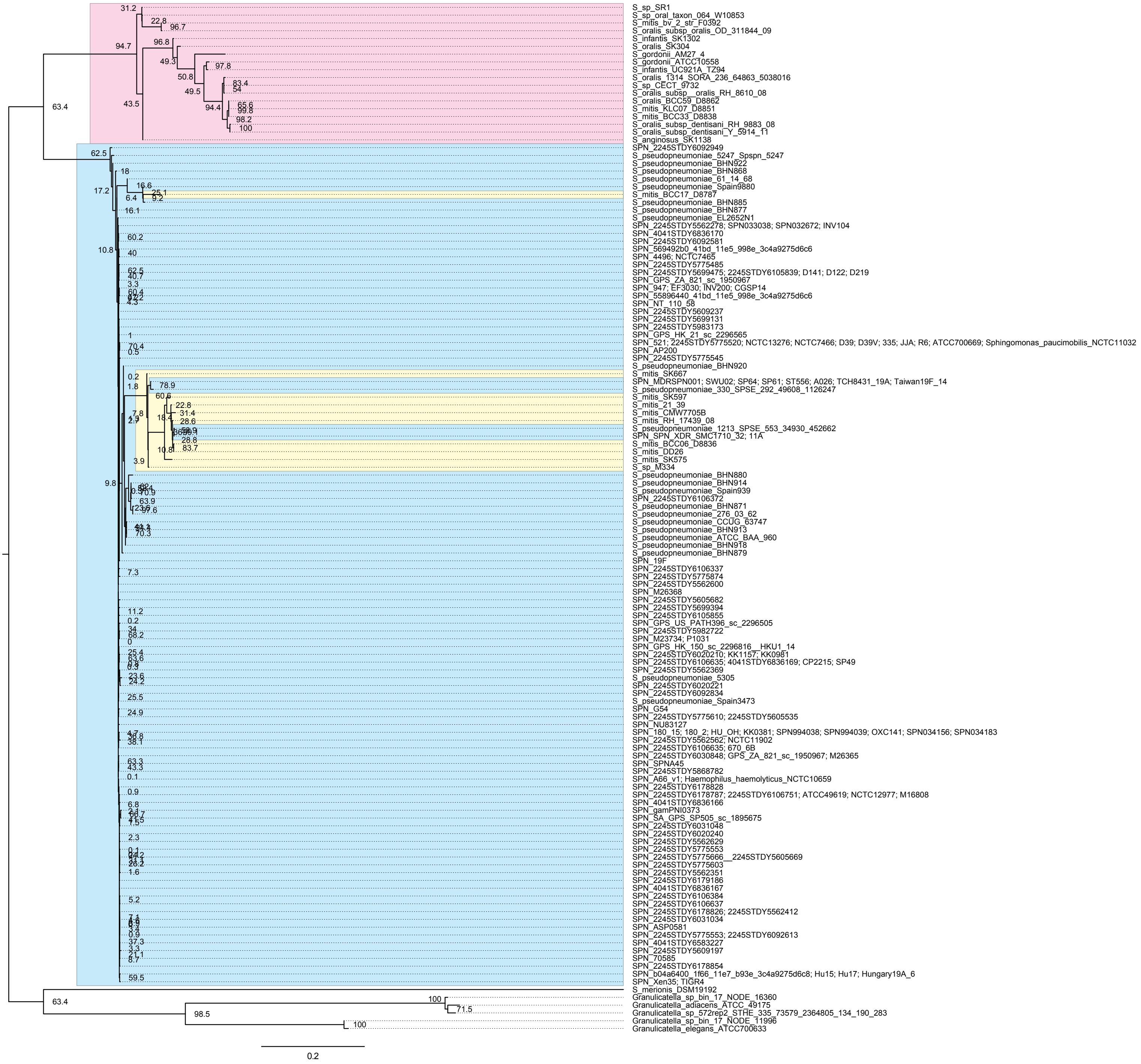

Supplementary Figure 1. Yamaguchi *et al.*

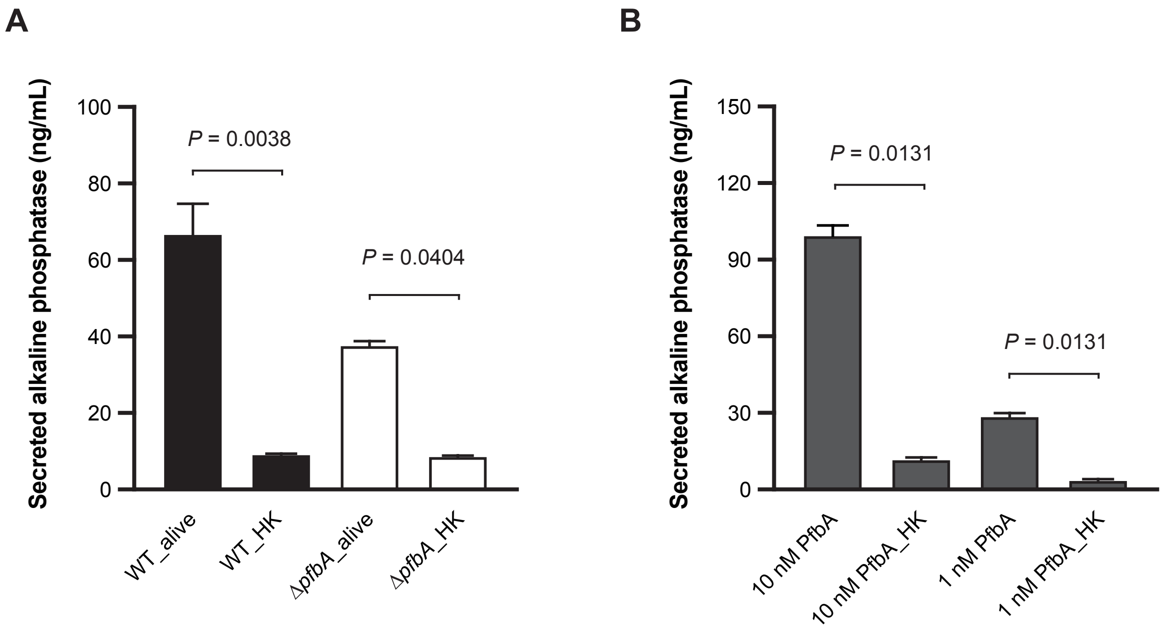

Supplementary Figure 2. Yamaguchi *et al.*

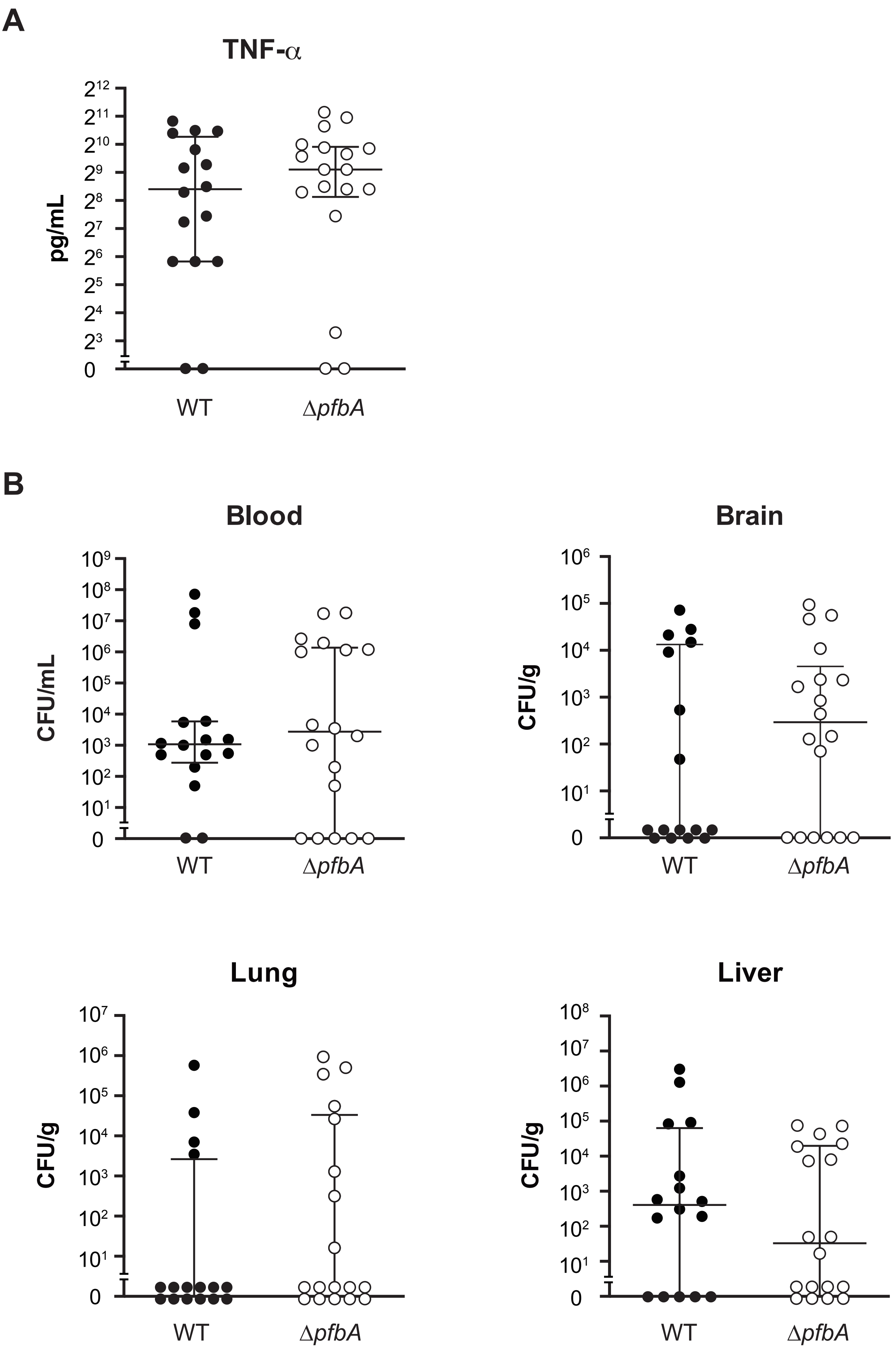

Supplementary Figure 3. Yamaguchi *et al.*

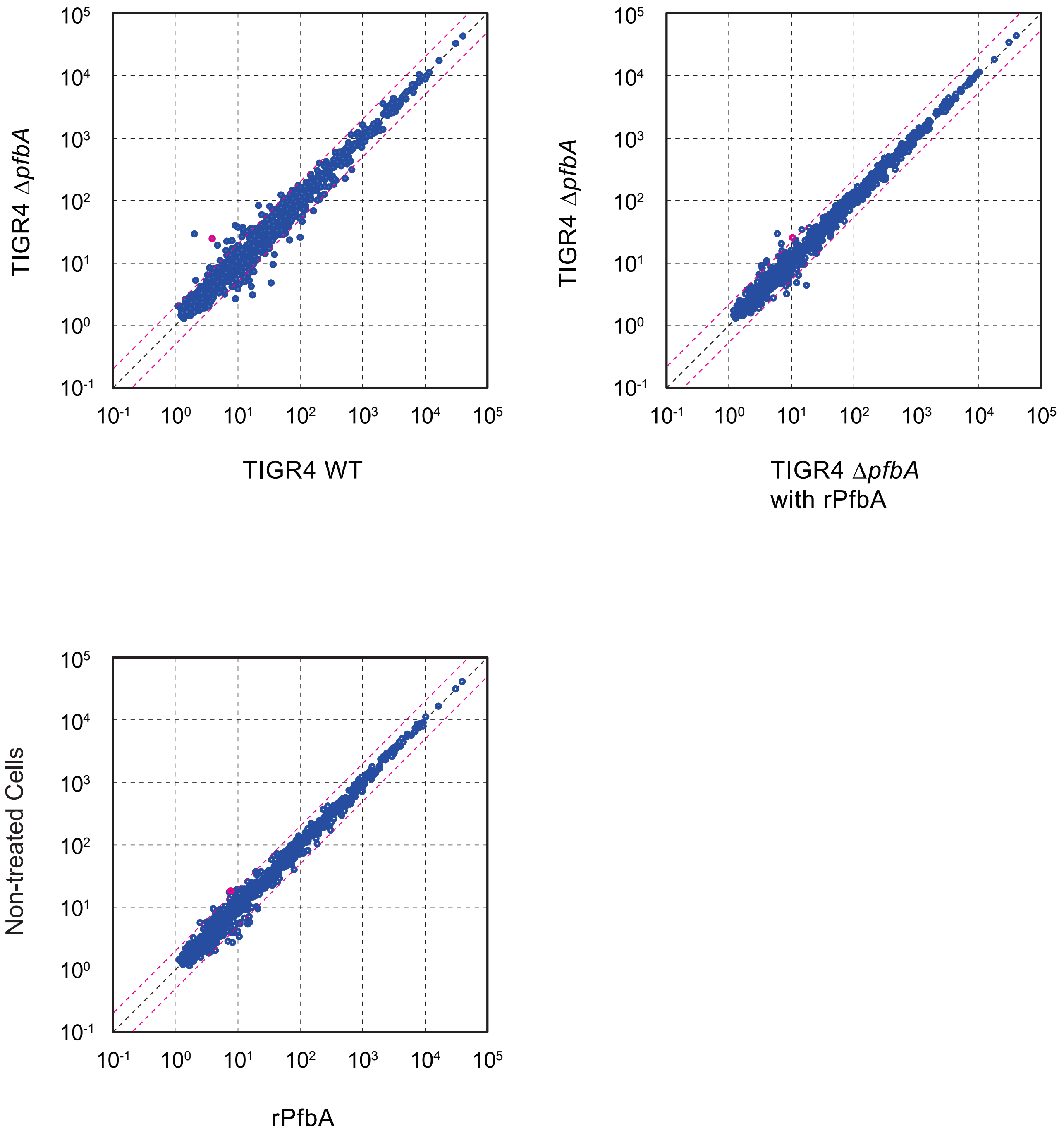

Supplementary Figure 4. Yamaguchi *et al.*
